# Multidrug and vancomycin resistance among clinical isolates of *Staphylococcus aureus* from different teaching hospitals in Nigeria

**DOI:** 10.4314/ahs.v17i3.23

**Published:** 2017-09

**Authors:** Olajuyigbe Olufunmiso, Ikpehae Tolulope, Coopoosamy Roger

**Affiliations:** 1 Department of Microbiology, School of Sciences and Technology, Babcock University, PMB 4005, Ilisan-Remo, Ogun State, Nigeria; 2 Department of Nature Conservation, Faculty of Natural Sciences, Mangosuthu University of Technology, Durban, South Africa

**Keywords:** Bacterial resistance, vancomycin resistant *S. aureus*, susceptibility studies, agar dilution

## Abstract

**Backgrounds:**

*Staphylococcus aureus* has emerged as a major public health concern because of the occurrence of multi-drug resistant strains. This study aimed at investigating the multi-drug and vancomycin resistance profile of *S. aureus* from different infection sites in some teaching hospitals in Nigeria.

**Methods:**

Swabs were collected from different infection sites from out-patients in three teaching hospitals from October 2015 to May, 2016. The antibiotic-susceptibility test was carried out with selected antibiotics usually administered anti-microbials in the treatment of infections in these hospitals. The prevalence of multi-drug and vancomycin resistance strains of *S. aureus* from clinical samples was determined using disk diffusion and agar dilution methods respectively.

**Results:**

The result showed (165)82.5% of the isolates were resistant to ≥3 antibiotics tested. They were highly resistant to ceftazidime 180(90%), cloxacillin 171(85.6%) and augmentin 167(83.3%), but susceptible to ofloxacin 150(75%), gentamicin 142(71.7%), erythromycin 122(61.1%), ceftriaxone 111(55.6%) and cefuroxime 103(51.7%). All the isolates from the HVS were all multidrug resistant strains. While (56)90.16% were multidrug resistant (MDR) in urine samples, followed by (8)88.89% MDR strains in sputum, (37)88.81% MDR strains in semen, (49)71.64% MDR strains in wounds and (6)60% MDR strains in ear swabs samples. Although (147)73.5% of the isolates were vancomycin susceptible *S. aureus* (VSSA), (30)15% were vancomycin intermediate resistant *S. aureus* (VISA) and (89)44.5% of the isolates were considered vancomycin resistant *S. aureus* (VRSA).

**Conclusions:**

The high percentage of the VRSA could have resulted from compromising treatment options and inadequate antimicrobial therapy. The implication, infections caused by VRSA would be difficult to treat with vancomycin and other effective antibiotics of clinical importance. Ensuring proper monitoring of drug administration will, therefore, enhance the legitimate role of vancomycin as an empiric choice for both prophylaxis against and treatment of *staphylococcal* infections.

## Introduction

*Staphylococcus aureus* is frequently found in the human respiratory tract and on the skin. It is estimated that 20% of the human population are long-term carriers of *S. aureus*^1^ whereas it is a transient normal flora of human skin and mucosal surfaces in 20 to 90% of healthy population. To establish its pathogenic potential, *S. aureus* produces toxin and extracellular membrane compounds.^4^ It produces various virulence factors including coagulase to clot plasma and coats the bacterial cells to probably prevent phagocytosis,^5^ hyaluronidase and DNAse to break down hyaluronic acid and DNA respectively to help in its systemic spread^6^ as well as staphylokinase to dissolve fibrin.^5^ While these virulence factors allow its attachment to host's cells, invade tissues and evade the host's immune system, Silva and Gandra^4^ indicated that enzymes like coagulase and catalase produced by *S. aureus* are responsible for the invasion of the immune system.

*S. aureus* infects wounds,^7^ cause ascending urinary tract colonization and infection^8^ and atopic dermatitis.^9^ While it is responsible for necrotizing pneumonia, skin and soft tissue infections, bacteraemia as well as food poisoning through enterotoxin production^10^–^12^ and may occur as commensals,^13^ this organism can infect tissues when the skin or mucosal barriers have been breached^9^, to cause infections associated with increased burden on healthcare resources^14^ in community and hospitals^15^. The unrestricted use of antibiotics and inadequate compliance to antibiotic regime along with inadequate surveillance for anti-microbial resistance are some of the imperative reasons accrued to the emergence of its highly resistant strains.^16^,^17^

Since the emergence of penicillin and methicillin resistant *S. aureus* strains in 1948 and 1961 respectively^18^,^19^ and virtually all strains of *S. aureus* are, today, resistant to natural penicillins, aminopenicillins and antipseudomonal-penicillins,^20^,^21^ it becomes necessary to find alternative antibiotics to treat *staphylococcal* infections.^22^ Consequently, vancomycin, a tricyclic glycopeptide antibiotic, is used to treat Gram-positive infections involving methicillin resistant *S. aureus* (MRSA).^23^,^24^ This antibiotic interferes with bacterial cell wall synthesis, as does penicillin, to lyse the cell.^25^ However, soon after its introduction, reduced susceptibility to vancomycin was reported in Japan by Hiramatsu.^26^ This was quickly followed by isolation of vancomycin intermediate resistant *S. aureus* (VISA) and vancomycin resistant *S. aureus* (VRSA) isolates from France,^27^ United Kingdom,^28^ Brazil,^29^ USA,^30^,^31^ Germany, 32 India^33^,^34^ and Belgium^35^,^36^ to confirm that the emergence of these strains is a global challenge. From patients treated with glycopeptides and in patients with suspected or confirmed MRSA, vancomycin intermediate and a few vancomycin-resistant strains have been isolated.^37^–^39^ While Assadullah et al.^33^ and Khadri and Alzohairy^40^ indicated that VRSA is not widely seen and a low level of resistance to vancomycin is being reported, the knowledge of the prevalence of VRSA and their antibiotic susceptibility pattern becomes fundamental in the selection of appropriate empirical treatment especially in hospital settings in the third world countries like Nigeria. This study, therefore, aimed at investigating the multi-drug and vancomycin resistance profile of *S. aureus* from different infection sites in some teaching hospitals in Nigeria. This is to detect VRSA as potential risk factor that could pose challenges to the effectiveness of anti-microbial therapy in the treatment of *staphylococcal* infections in developing countries like Nigeria.

## Materials and methods

Samples were collected from 200 patients attending three teaching hospitals in Ogun State, Nigeria. These patients were being treated at out-patient Units of Babcock University Teaching Hospital, Ilisan-Remo, Olabisi Onabanjo Teaching Hospital, Sagamu and Federal Medical Center, Idi-Aba, Abeokuta, all in Ogun State, Nigeria from October 2015 to May 2016. Patients being treated with systemic antibiotics in the last 4 weeks were excluded. The test samples collected were taken by carefully rolling swabs saturated with sterile peptone water in the different infection sites from different teaching hospitals in Nigeria. The swabs were tightly sealed and immediately transported to the laboratory. The collected infection swab sticks were streaked on mannitol salt agar (MSA) and nutrient agar which were incubated overnight at 37°C for 24–48 h.^41^ The bacterial colonies were subjected to established procedures such as Gram staining, microscopic appearance, colony morphology and biochemical tests such as tube DNase, catalase and coagulase tests for the characterization of the strains.^42^–^44^

## Antibiograms of the isolates using multi-disc antibiotics

Each of the isolates was standardized using colony suspension method. Each strain's suspension was matched with 0.5 McFarland standards to give a resultant concentration of 1.5 × 10^6^ cfu/ml. The antibacterial activity was determined using agar diffusion assay technique according to the modified Kirby-Bauer diffusion technique^43^ by swabbing the Mueller-Hinton agar (MHA) (Oxoids UK) plates with the adjusted overnight culture of each of the test isolates. Multi-discs (Abtek) containing different antibiotics including ofloxacin (5 µg), augmentin (30 µg), ceftazioime (30µg), cefuroxine (30 µg), gentamicin (10 µg), ceftrioxone (30 µg), erythromycin (5 µg) and cloxacillin (5 µg) were aseptically placed on the inoculated agar plates and incubated at 37°C for 24 h. After 24 h of incubation, the plates were examined for inhibition zones.^45^ The diameter of the inhibition zones produced by each antibiotic disk was measured to the nearest millimeter, recorded and interpreted using the Clinical and Laboratory Standard Institute Zone Diameter Interpretative Standards.^46^ Each bacterial isolate was classified as susceptible (S), intermediate (I) and resistant (R) to antibiotics according to the zone diameter interpretation standard recommended by the Clinical Laboratory Standards Institute.^47^

## Susceptibility of the isolates to vancomycin

The susceptibility of the different strains of *S. aureus* to vancomycin was further determined by agar dilution method using CLSI guidelines.^48^ Here, gradient plates of Mueller Hinton agar were prepared with different concentrations, 1 µg/ml, 2 µg/ml, 4 µg/ml, 8 µg/ml and 16 µg/ml, of the vancomycin by dissolving vancomycin tablets (Mast Diagnostics, Mast Group Ltd., Merseysidem UK, Lot: 311380, Exp: 2016 — 06) in 200 ml of sterilized molten agar maintained at a temperature of 50°C. The conical flasks containing the vancomycin tablets were then mixed gently till the tablets were completely dissolved. The antibiotic-containing agar was then dispensed aseptically into Petri dishes labeled according to the various concentrations of the vancomycin prepared and allowed to solidify. 0.5 McFarland equivalent inoculums prepared using 18 h old culture to give a resultant concentration of 1.5 × 10^6^ cfu/ml was inoculated by streaking and stabbing the concentration gradient vancomycin-containing agar plates. The plates were incubated overnight at 35°C before being assessed for visible growth. Appearance of growth indicated vancomycin resistance.^49^ Hence, the points of streaking and stabbing that showed bacterial growth were referred to as being vancomycin resistant while the points that did not show bacterial growth were referred to as vancomycin susceptible.

## Results

**Figure 1:**
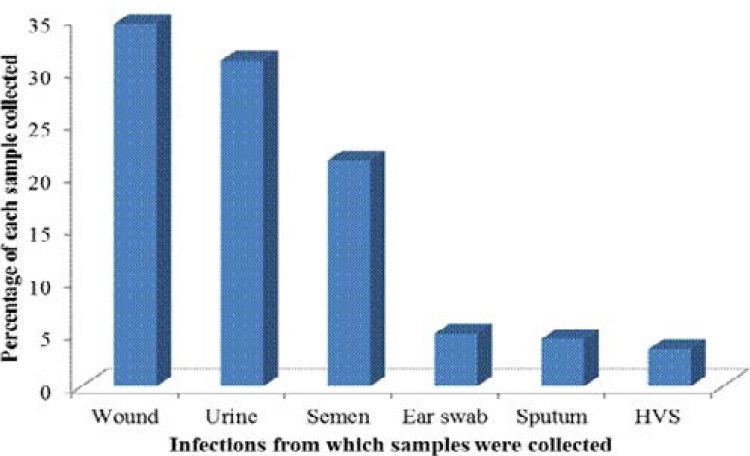
Percentage distribution of isolates from different infection sources

A total number of 200 clinical strains of *S. aureus* were isolated from wound, urine, semen, ear swabs, sputum and high vaginal swabs (HVS) and characterized from different teaching hospitals in Lagos state. From the sample distribution, the highest incidence of *S. aureus* was in wounds 69(34.5%) followed by urine 62(31%), semen 42(21%), ear swabs 10(5%), sputum 9(4.5%) and HVS 7(3.5%) as shown in Figure 1.

**Figure 2:**
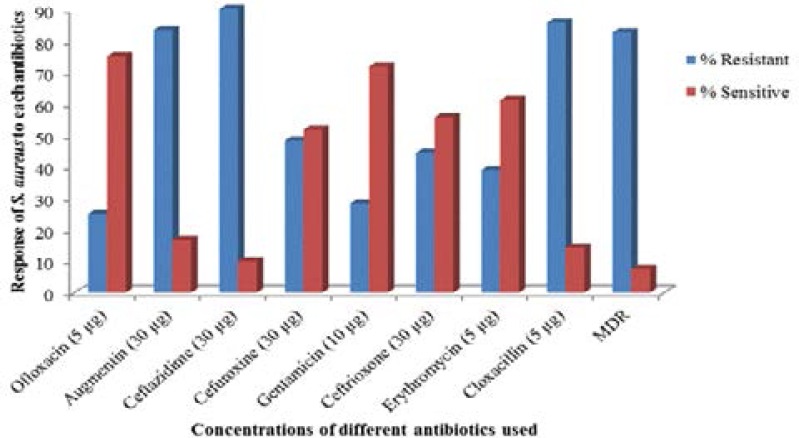
Resistance and Sensitivity profiles of *Staphylococcus aureus* to different antibiotics

Though these isolates were highly resistant to ceftazidime 180(90%), Cloxacillin 171(85.6%) and augmentin 167(83.3%), (150)75% of the isolates were susceptible to ofloxacin, followed by gentamicin 143(71.7%), erythromycin 122(61.1%), ceftriaxone 111(55.6%) and cefuroxime 103(51.7%) in a descending order. However, (165)82.5% of the isolates exhibited multidrug resistance by being resistant to ≥3 of the test antibiotics as shown in Figure 2.

From the susceptibility of these isolates to different concentrations of vancomycin used in this study as shown in Table 1, (97)48.5% were susceptible to all the concentrations of the vancomycin used while (34)17% were resistant to all the concentrations of the vancomycin used. Considering the number of strains susceptible at each concentration used, (153)76.5%, (149)74.5%, (137)68.5%, (123)61.5% and (111)55.5% of the *S. aureus* isolates were susceptible at 1 µg/ml, 2 µg/ml, 4 µg/ml, 8 µg/ml and 16 µg/ml respectively. Considering the number of isolates susceptible at concentrations less than or equal to each of the concentration used, (147)73.5%, (15)7.5% and (15)7.5% of the isolates were susceptible at concentrations of ≤2 µg/ml, ≤4 µg/ml and ≤8 µg/ml respectively but different percentages of isolates were resistant at higher concentrations above the respective concentrations at which they were susceptible. Considering the number of isolates that were initially resistant to vancomycin but later became susceptible to this antibiotic, (2)1% of the isolates were resistant at 1 µg/ml but susceptible to other concentrations, (5)2.5% of the isolates were resistant at ≤2 µg/ml but susceptible to all the other concentrations and (3)1.5% were resistant at ≤8 µg/ml but susceptible only at 16 µg/ml but none of the isolates was resistant at concentration before 4 µg/ml.

**Table 1:** The susceptibility of *Staphylococcus aureus* strains to different concentrations of vancomycin antibiotic

Susceptibility of *Staphylococcus aureus* strains to different concentrations of vancomycin antibiotic							
	
S/N	*S. aureus*	Source	1 µg/ml	2 µg/ml	4 µg/ml	8 µg/ml	16 µg/ml
1.	MDR	Wound	−	−	−	−	−
2.	MDR	Wound	+	+	+	+	+
3.	MDR	Wound	−	−	+	+	+
4.	MDR	Wound	−	−	−	+	+
5.	SS	Wound	−	−	−	+	+
21.	MDR	Wound	−	−	−	+	+
22.	MDR	Wound	−	−	−	−	−
23.	SS	Wound	−	−	−	−	−
24.	MDR	Wound	−	−	−	−	−
25.	MDR	Wound	−	−	−	−	+
26.	MDR	Wound	−	−	−	−	+
27.	MDR	Wound	−	−	−	−	−
28.	SS	Wound	−	−	−	−	−
29.	MDR	Wound	+	+	+	−	−
30.	SS	Wound	−	−	−	−	−
31.	MDR	Wound	−	−	−	−	−
32.	SS	Wound	−	−	−	−	−
33.	MDR	Wound	−	−	−	−	−
34.	MDR	Wound	−	−	−		
51.	MDR	Wound	+	+	+	+	+
52.	MDR	Wound	−	−	−	−	−
53.	MDR	Wound	−	−	−	−	+
54.	SS	Wound	−	−	−	−	−
55.	MDR	Wound	+	+	+	+	+
56.	SS	Wound	−	−	−	−	+
57.	MDR	Wound	−	−	+	+	+
58.	MDR	Wound	−	−	−	−	−
72.	MDR	Semen	−	−	−	−	−
73.	SS	Semen	−	−	−	−	−
74.	MDR	Semen	+	+	+	+	+
75.	MDR	Semen	−	−	+	+	+
76.	MDR	Semen	+	+	+	+	+
77.	MDR	Semen	+	+	+		
94.	MDR	Semen	+	+	+	+	+
95.	MDR	Semen	+	+	+	+	−
96.	MDR	Semen	−	−	+	+	+
97.	MDR	Semen	+	+	+	+	+
98.	MDR	Semen	−	−	−	−	−
112.	MDR	Semen	−	−	−	−	−
113.	MDR	Urine	−	−	−	+	+
114.	MDR	Urine	−	−	−	+	
130.	MDR	Urine	+	+	+	−	−
131.	MDR	Urine	−	−	−	−	−
132.	MDR	Urine	−	−	−		
151.	MDR	Urine	−	−	−	−	−
152.	SS	Urine	−	−	−	−	−
153.	MDR	Urine	−	−	−	−	−
154.	MDR	Urine	−	−	−	+	+
155.	SS	Urine	−	−	−	−	−
165.	SS	Urine	−	−	−	−	−
166.	MDR	Urine	−	−	−	+	+
167.	MDR	Urine	−	−	−	−	
182.	SS	Sputum	−	−	−	−	−
183.	MDR	Sputum	−	−	−	−	−
184.	MDR	Ear-swab	−	−	−	−	−
193.	SS	Ear-swab	−	−	−	−	−
194.	MDR	HVS	−	−	+	+	+
195.	MDR	HVS	−	−	−	+	+
196.	MDR	HVS	−	−	−	−	−
197.	MDR	HVS	−	−	−	−	−
198.	MDR	HVS	+	+	+	+	−
199.	MDR	HVS	−	−	−	−	−
200.	MDR						

According to the Clinical Laboratory Standards Institute (CLSI, formerly NCCLS), *S. aureus* isolates for which vancomycin MIC are 4–8 µg/ml are classified as vancomycin-intermediate (VISA), and isolates for which vancomycin MIC's are greater than 8 µg/ml are classified as vancomycin-resistant.^50^ From case definition of Kluytmans et al.^51^ indicating vancomycin MIC of ≤2 µg/ml as vancomycin-susceptible *S. aureus* (VSSA), vancomycin MIC = 4–8 µg/ml as vancomycin-intermediate susceptible *S. aureus* (VISA) and vancomycin MIC ≥ 16 µg/ml as vancomycin-resistant *S. aureus* (VRSA), (147)73.5% of the isolates were vancomycin susceptible *S. aureus* (VSSA). Combining those isolates susceptible at concentrations ≤4 µg/ml and those of susceptible at ≤8 µg/ml, (30)15% of the isolates were considered vancomycin intermediate resistant *S. aureus* (VISA). However, (34)17% of the isolates that were resistant to all the concentrations of the vancomycin used were considered VRSA in addition to (55)27.5% of the isolates that were resistant to vancomycin at the concentration of 16 µg/ml. Hence, (89)44.5% of the isolates were considered VRSA. Considering the distribution of *S. aureus* on the basis of their susceptibility to vancomycin with respect to isolates from the different sampling sources, (180)90% of the *S. aureus* from ear samples were susceptible to vancomycin while (20)10% of the isolates were vancomycin intermediate resistant *S. aureus* (VISA) and no vancomycin resistant *S. aureus*. In HVS, VSSA was (4)57.14%, VISA was (2)28.5% and VRSA was (1)14.3%.

**Figure 3:**
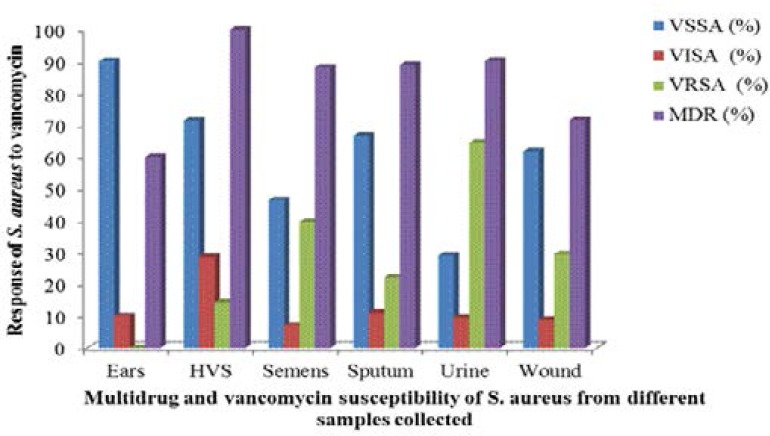
Percentage distribution of multi-drug and vancomycin susceptibility of *S. aureus* in each infection samples

In semen samples, VSSA was (22)53.5%, VISA was (3)7% and VRSA was (17)39.5%. In sputum samples, VSSA was (6)66.7%, VISA was (1)11.1% and VRSA was (2)22.2%. While VSSA was (18)29.0%, VISA was (6)9.7% and VRSA was (38)61.29% in urine samples, wound samples had VSSA 43(61.8%), VISA 6(8.8%) and VRSA 20(29.4%). Invariably, the percentages of VRSA in the various samples collected varied in a descending order from urine samples having VRSA 38(61.29%), followed by semen 17(39.5%) > wound 20(29.4%) > sputum 2(22.2%) > HVS 1(14.3%) > ear swabs 0(0%). While the isolates from the HVS were all multi-drug resistant strains, (59)90.2% of isolates from the urine samples were multi-drug resistant, followed by (8)88.9% MDR strains from sputum, (37)88.81% MDR strains from semen, (49)71.64% MDR strains from wounds and (6)60% MDR strains from ear swabs as shown in Figure 3.

## Discussion

*Staphylococcus aureus* has been one of the most problematic nosocomial pathogens and a major threat to human health worldwide due to its anti-microbial resistance, infectivity and possession of virulence factors^52^,^53^ as well as its ability to repeatedly acquire resistance to overcome the challenges presented by the new anti-*staphylococcal* antibiotics. 54 Although vancomycin is the main antimicrobial agent available to treat serious *staphylococcal* infections, especially those of MRSA, a decrease in vancomycin susceptibility of *S. aureus* and isolation of vancomycin intermediate and resistant *S. aureus* from many countries have been reported.^55^–^57^ Since its first being reported in 1997, the threat of vancomycin resistance in *S. aureus* has been the topic of intensive research, discussion and cause for alarm in the health care community.^58^ There is widespread concern that vancomycin-resistant *S. aureus* poses, by far, the greatest risk to patients, given the virulence of the organism.

In this study, the prevalence of VRSA was found to be (89)44.5% of the investigated *S. aureus* isolated. However, the (89)44.5 % vancomycin resistance rate of *S. aureus*, in this study, was higher than that of 21% reported by Flamm et al.^59^ in Nepal, 3.6% reported in Iran,^60^ 40% reported by Mimejad et al.^61^ in Iran and the 16.4% reported by Godebo et al.^62^ in Ethiopia. Although these variations in the degree of resistance are geographically based, these varied degrees of resistance to vancomycin have resulted in an increasing concern about its therapeutic effectiveness in serious *staphylococcal* infections. While the determination of the antimicrobial susceptibility is crucial for an optimal therapy, for epidemiological purposes and for infection control measures,^60^,^63^ the treatment of the *S. aureus* infections has become problematic because of the emergence of resistance to methicillin, vancomycin and other antibiotics.^60^,^64^

In agreement with De Lassence et al.^65^ who indicated that VRSA tend to be multi-drug resistant against a large number of currently available anti-microbial agents, compromise treatment options and increase the likelihood of inadequate anti-microbial therapy and a resultant increase in morbidity and mortality, VRSA, a trait assigned to *S. aureus* strains with vancomycin minimum inhibitory concentration greater than 8 µg/ml,^66^,^67^ showed high percentages of resistance to a wide range of anti-microbial agents including augmentin 167(83.3%), cloxacillin 171(85.6%) and ceftazidime 180(90%). Consequently, treatment of *Staphylococcus* infections will become more difficult because (165)82.5% of the strains, in this study, were resistant to ≥3 antibiotics tested at the same time.^68^ As the frequency of antibiotic-resistant bacteria among countries is proportional to their relative rates of antibiotic use,^69^,^70^ a never-ending need to produce and market costlier new antibiotics to treat progressively more resistant infections is inevitable.^71^

As the case may be in Nigeria and some other developing countries, virtually all drugs are sold in drug stores called “Chemists” in the local parlance without obtaining antibiotic sensitivity test results from the medical laboratories or prescriptions from clinicians. These factors, according to Yah et al.^72^, increase the rate of drug abuse and consequently increase the rate of development of bacterial resistance to antibiotics in a geometric rate higher than that in developed countries. In this study, the presence of VISA may be an important indicator of the insidious decline of the clinical effectiveness of vancomycin in the hospitals or injudicious use of vancomycin in hospitals for wrongly diagnosed or false positive MRSA. While (34)17% of the isolates were resistant to MIC ≥16 µg/ml of the vancomycin antibiotic showed a fast increasing rate of development of vancomycin resistant *S. aureus* especially among clinical isolates, having (89)44.5% VRSA is an indication that *S. aureus* has become more resistant to vancomycin in comparison to other reports. This may, probably, pose a big problem towards its use as the ultimate drug against MRSA. These isolates may have acquired resistance by mutation and thickening of cell wall due to accumulation of excess amounts of peptidoglycan. 73,74 The cell wall thickening may have caused vancomycin molecules to become trapped in the outer layers of the cell wall, clog the peptidoglycan meshwork and form physical barriers limiting its access to the cytoplasmic membrane where the functional targets of vancomycin are located.^75^

In this study, that the percentages of VRSA varied in a descending order from urine samples having VRSA 38(61.26%), followed by semen 17(39.5%) > wound 20(29.4%) > sputum 2(22.22%) > HVS 1(14.29%) > ears (0%) is contrary to the report of Dhand et al.^76^ who found no VRSA, VISA and VSSA in 250 *S. aureus* from clinical samples. On the other hand, while VRSA (4.7%), VISA (9.3%) and VSSA (86.0%) were reported by Ilang et al.^77^, 26.7% of VRSA in post-operative pus samples^78^ and 36.1% of VISA in blood and body fluids^79^ were reported. These differences might be due to prolonged antibiotic treatment of severely sick patients, who generally have longer hospital stays, resulting in enhanced selection pressure. Therefore, prolonged use of antibiotics and prolonged hospitalization are other important factors making hospitals an ideal place for transmission and perpetuation of VRSA.^80^

In conclusion, this study shows that there is a high prevalence of vancomycin-resistant *S. aureus* (VRSA) amongst isolates from the clinical samples investigated. The VRSA were multi-drug resistant against a large number of currently available anti-microbial agents. The high percentage of the VRSA could have resulted from compromising treatment options and inadequate anti-microbial therapy, a lack of sufficient knowledge on the danger of the wrong use of antibiotics, high proximity to a large number of unlicensed drug vendors and the inappropriate use of broad spectrum antibiotics in the medical practice. Efforts should, therefore, be made in ensuring proper monitoring of drug administration and its use to prevent drug misuse and abuse as well as to prevent or reduce the rate of anti-microbial resistance amongst clinical pathogens. These will, therefore, enhance the legitimate role of vancomycin as an empiric choice for both prophylaxis against and treatment of *staphylococcal* infections.
